# Lobectomy in traumatic brain injury patients with intracerebral hemorrhage and delayed contusion

**DOI:** 10.5249/jivr.vo112i2.1180

**Published:** 2020-07

**Authors:** Shahrokh Yousefzadeh-Chabok, Mohammad Safaei, Ehsan Kazemnejad, Davoud Mahmoudi, Sasan Andalib

**Affiliations:** ^*a*^ Guilan Road Trauma Research Center, Poursina Hospital, School of Medicine, Guilan University of Medical Sciences, Rasht, Iran.; ^*b*^ Department of Neurosurgery, Poursina Hospital, School of Medicine, Guilan University of Medical Sciences, Rasht, Iran.; ^*c*^ Neuroscience Research Center, Department of Neurosurgery, Poursina Hospital, School of Medicine, Guilan University of Medical Sciences, Rasht, Iran.; ^*d*^ Research Unit of Clinical Physiology and Nuclear Medicine, Department of Nuclear Medicine, Odense University Hospital, Faculty of Health Sciences, University of Southern Denmark, Odense, Denmark.; ^*e*^ Research Unit of Psychiatry, Department of Psychiatry, Department of Clinical Research, Faculty of Health Sciences, University of Southern Denmark, Odense, Denmark.; ^*f*^ BRIDGE-Brain Research-Inter-Disciplinary Guided Excellence, Department of Clinical Research, University of Southern Denmark, Odense, Denmark.

**Keywords:** Brain Lobectomy, Traumatic Brain Injury, Glasgow Outcome Scale, Glasgow Comma Scale

## Abstract

**Background:**

TBI, standing for Traumatic Brain Injury, is a leading cause of death worldwide; nonetheless, data on its management has hitherto been sparse. In view of the fact that brain lobectomy is a contentious issue in the management of TBI, we set out the current study to assess the mortality rate and outcomes of TBI with delayed contusion or Intracerebral Hemorrhage (ICH) undergoing lobectomy.

**Methods:**

We evaluated 135 TBI patients with delayed contusion or ICH undergoing brain lobectomy from 2001 to 2013. Withal, the mortality and Glasgow Outcome Scale (GOS) and Glasgow Comma Scale (GCS) rates were assessed in these patients and the association in between was sought.

**Results:**

The TBI patients undergoing brain lobectomy (77% male versus 23 % female) had a mean age of 43.4±20.3 years and experienced a survival rate of 62.2% (71% in females versus 60% in males). Favorable GOS was observed in 53% of male patients, compared with 27% in the females. Age was demonstrated to significantly affect the mortality rate (p=0.0001). Initial GCS score was associated with GOS as 79.1% of the survived patients with a GCS of higher than 9 on admission were discharged with favorable GOS.

**Conclusions:**

The evidence from the present study indicates that lobectomy can be an acceptable surgical procedure in management of TBI patients with delayed contusion or ICH.

## Introduction

TBI, which refers to traumatic brain injury, arises from mechanical external force and disrupts the normal brain function. It can give rise to concussion, contusion, intracranial hemorrhage, intracerebral hemorrhage (ICH), and epidural and subdural hematomas; be that as it may, a broad spectrum of long-term symptoms and disabilities may exist in TBI. Intracranial pressure (ICP) increase and its evaluation is important in TBI.^1^ Patients with TBI are treated in order to reduce brain edema. Moreover, it is said that lobectomy and lesionectomy can decrease secondary insult, ICP, brain herniation risk and improves Glasgow Coma Scale (GCS) and other neurological symptoms. Thus, it may decrease hospitalization period and treatment cost. The literature has been flooded by evidence of the effect of decompressive craniectomy on the patients with severe TBI with contusion or ICH;^2-6^ nevertheless, a few studies only evaluated the impact of lobectomy on these patients.^7,8^ The aim of the present study is to assess the mortality rate and outcomes of TBI patients with ICH or delayed contusion undergoing lobectomy.

## Materials and Methods

The present cross-sectional study was approved by local research review panel and carried out at Department of Neurosurgery, Poursina Hospital, School of Medicine, Guilan University of Medical Sciences. Patients with TBI with delayed contusion or ICH undergoing lobectomy between 2002 and 2014 (age: 18-70 years) were included. Exclusion criteria were a history of TBI and neurological, lung and cardiovascular disorders, and anticoagulant treatment. Glasgow Outcome Scale (GOS) was scored for all the patients. Favorable and unfavorable outcomes were defined as a GOS of 4-5 and 2-3, respectively. On admission, data regarding iris response to light (number of responding iris and dilatation), systolic blood pressure (hypertension, normal blood pressure, hypotension) and respiratory conditions (being on ventilation or not), side of brain injury, distance of the accident location from hospital (less and more than 50 km), shifts of admission (morning, evening, and afternoon), various TBIs, and NICU hospitalization, primary and secondary insults, injured brain lobes, time of GCS decrease, and time between GCS decrease and surgery were recorded. The follow-up was made 6 months after the brain lobectomy. Data were analyzed by SPSS (version 21), more specifically using independent t-test, chi square test, and Fisher’s exact test. A P-value of less than 0.05 was considered as statistically significant.

**Surgical technique consideration**

Surgical technique depends on the location of the lesion. In the right side of the brain, which is non-dominant side, frontal lobe for 3-4 cm and temporal lobe for 5-6 cm to Labbe vein area can be resected. Howbeit, in the left side, only contused and amorphic areas can be resected. Such resection can be carried out not more than 3-4 cm only in superior temporal gyrus and anterior aspect of middle fossa. In the left frontal lobe, it is only allowed for a maximum size of 2.5 cm.

## Results

**Figure 1:**
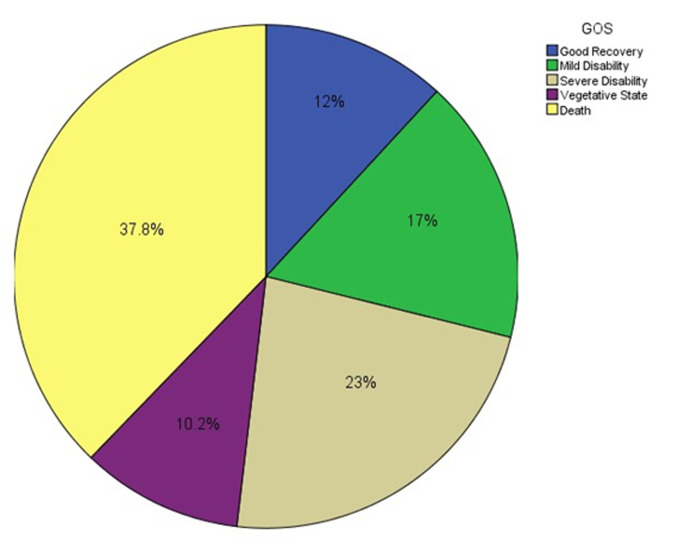
Proportion of various levels of GOS in the patients with TBI with delayed contusion or ICH undergoing lobectomy.

In the present study, 205 TBI patients with delayed contusion or ICH undergoing brain lobectomy were initially selected; howbeit, after excluding 70 patients, we ultimately evaluated 135 patients. The mean±SD age of the patients was 43.4±20.3 (range=19-85) years. Males and females accounted for 77% (n=104) and 23% (n=31) of the patients, respectively. The survival rate in these TBI patients was 62.2% (n=84) after brain lobectomy. Sixty percent (n=62) of males and 70% (n=22) of females were survived after the lobectomy. GCS levels, that is to say, death, persistent vegetative state, severe disability, moderate disability, and good recovery were observed in 37.8 (n=51), 10.2 (n=14), 23 (n=31), 17 (n=23), 12 (n=16) percent of the operated patients (Figure 1).Table 1 illustrates that the TBI patients had various primary and secondary pathologies and injured brain lobes. The best prognosis was seen in those with only contusion with a survival rate of 81% and a favorable GOS in 76.5%. The frontal lobe was the most prevalent injured brain region; albeit TBI patients with such injury experienced the best GOS (61%). Of the patients who had a GCS decrease after Day 4, 11.1% showed a favorable outcome after the operation.

**Table 1:** Primary and secondary insults, injured brain lobes, time of GCS decrease, and time between GCS decrease and surgery in TBI patients (Note: ICH: Intracerebral hemorrhage; DF: Depressed fracture; EDH: Epidural hematoma; SDH: Subdural).

		N(%)
**Primary insult**	ICH	44(32.6)
****	Contusion	21 (15.6)
****	ICH+contusion	36(26.7)
****	ICH+contusion+DF	16(11.8)
****	ICH+contusion+EDH	2(1.5)
****	ICH+contusion+SDH	16(11.8)
**Secondary insult**	No change	18(13.3)
****	Increase in hematoma volume	99(73.4)
****	New hematoma	18(13.3)
**Injured brain lobe**	Frontal	50(37.0)
****	Temporal	19(14.1)
****	Parietal	3 (2.2)
****	Occipital	0(0)
****	Frontal and temporal	29(21.5)
****	Temporal and parietal	30(22.2)
****	Parietal and occipital	0(0)
****	Temporal, parietal, and occipital	2 (1.5)
****	Frontal, temporal, and parietal	2(1.5)
**Time of GCS decrease**	Day 1	111(82.2)
****	Day 2-4	15(11.1)
****	After Day 4	9(6.7)
**Time between GCS decrease and surgery**	Shorter than 2 hours	40(29.6)
****	2-12 hours	71 (52.6)
****	Longer than 12 hours	24(17.8)

**Figure 2:**
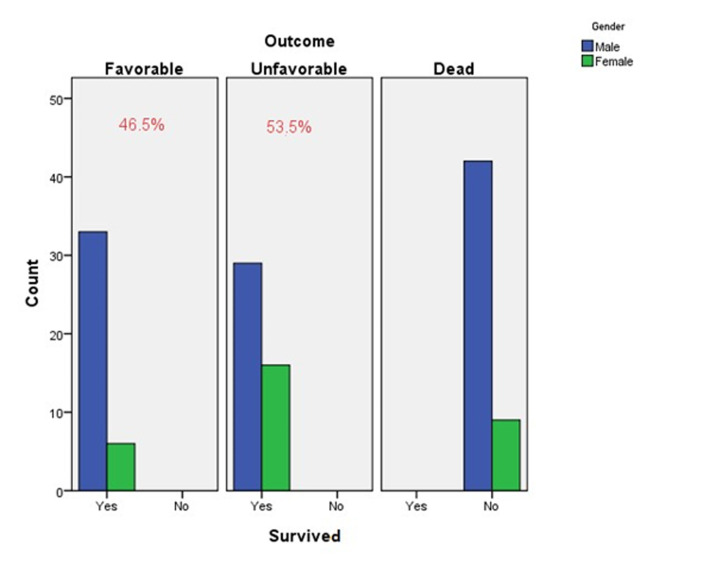
Favorable and unfavorable GOS in the survived TBI patients undergoing brain.

Of the patients who survived after operation, favorable GOS was seen in 39 (46.5%), including 33 males and 6 females. There was also unfavorable GOS in 45 survived patients (53.3%), including 29 males and 16 females. (Figure 2)

Gender was found to be associated with GOS (P=0.04) in the TBI patients. Moreover, age was significantly associated with both the mortality rate and the outcome (P=0.001). In contrast to mortality rate, there was an association between GOS and initial iris response to light (P=0.001). Systolic blood pressure and respiratory condition on admission were seen to be associated with GOS with a P value of 0.01 and 0.001, respectively, but not mortality rate. Lesion side was not associated with the mortality rate (P=0.30) and GOS (P=0.40). Mortality rate was not associated with distance of the accident location from hospital (P= 0.44), admission according to shifts (P=0.98), various traumatic lesions (P= 0.59), and hospitalization in Neurosurgery Intensive Care Unit (NICU) (P= 0.74). GOS was not associated with distance of the accident location from hospital (P= 0.17), shifts of admission (P= 0.98), various TBIs (P= 0.85), and NICU hospitalization (P= 0.78). The initial GCS was significantly associated with the GOS (P=0.001), notwithstanding the mortality rate (P=0.64) (Table 2). Initial GCS was correlated with outcome as 79.1% of the survived patients with a GCS of higher than 9 on admission were discharged with favorable GOS. 

**Table 2:** association initial GCS with the mortality and GOS in TBI patients undergoing brain lobectomy.

		GCS≥9	GCS<9	value
		N(%)	N(%)	
**Mortality**	Survived	41 (62.1)	43 (62.3)	0.645
	Death	25 (37.9)	26 (37.7)	
**GOS**	Favorable	25 (37.9)	34 (49.3)	0.001
	Non-favorable	36 (54.5)	9 (13)	
	Death	25 (37.9)	26 (37.7)	

## Discussion

TBI can be surgically treated by decompressive craniectomy^9^ or brain lobectomy. In comparison with decompressive craniectomy, lobectomy is an invasive procedure. Hence, a bulk of studies have focused on the outcomes of craniectomy in TBI patients and to the best of our knowledge, there are only two studies evaluating outcomes of TBI after brain lobectomy. The first study was performed by Litofsky et al.^7^ evaluating a small number of American TBI patients (n=20) with a mean age of 34 years (range:19-59 years) with a follow-up of 4 years. Another American study was carried out by Oncel et al.^8^ evaluating 183 TBI patients, most of which were males (80%), with a mean age of 36.3 years with a follow-up of 13 years. In the present study, age was shown to affect both the mortality rate and GOS, which corroborated findings of Litofsky et al.^7^ In comparison with aforementioned studies, we observed a low survival rate in our patients after lobectomy. In our study, favorable GOS (46.5%) reached almost the same level as that reported in the study of Oncel et al. ^8^ and initial GCS score was associated with GOS as 79.1% of the survived patients with a GCS of higher than 9 on admission were discharged with favorable GOS after brain lobectomy. This result was in agreement with the findings of Litofsky et al.^7^ indicating that patients with a mean GCS of 6.4 experienced unfavorable outcome and those with a mean GCS of 9.7 showed favorable outcome on discharge. Oncel et al.^8^ also found that TBI patients with GCS of less than 8 experienced a high mortality rate (33%), compared with those with GCS of higher than 8 (23%). Moreover, an association between GOS and initial iris response to light was detected in our study, which was consistent with the results of Litofsky et al.^7^ (p <0.04). We found that respiratory condition on admission was associated with GOS. We also detected that systolic blood pressure on admission was associated with GOS in the studied patients. Similarly, Oncel et al. ^8^ reported that half of the patients with hypotension owing to TBI passed away after the surgery. This may be due to the fact that hypotension and deteriorated respiratory condition bring about secondary insult in the brain after TBI. ICH, ICH with contusion, and ICH with hematoma constituted the most important pathology of the TBI in our study; albeit the best prognosis was seen in those with only contusion with a survival rate of 81% and a favorable GOS in 76.5%. The frontal lobe was the most prevalent injured brain region; howbeit, TBI patients with such injury experienced the best GOS. The findings of Litofsky et al. ^7^ do not support our results indicating that most of the patients with a GCS decrease after Day 4 fail to show a favorable outcome after brain lobectomy. In the present study, mortality rate was not associated with distance of the accident location to hospital, admission shifts, various TBIs, and NICU hospitalization. GOS was not associated with distance of the accident location to hospital, admission according to shifts, various traumatic lesions, and hospitalization in NICU. A limitation of our study is the sample size; therefore, we suggest that future studies with larger sample size is carried out in this regard. Another limitation is the cross-sectional nature of the present study. Prospective future studies are thus recommended. To draw a conclusion, the evidence from the current study suggests that lobectomy may be an acceptable surgical procedure in TBI patients with delayed contusion or ICH. Findings of the present study, along with those of the future study will be important for policy makers and departments of neurosurgery to consider the technique for TBI patients with delayed contusion or ICH. 

**Acknowledgment:**

We thank Guilan Road Trauma Research Center, Poursina Hospital, Guilan University of Medical Sciences, Rasht, Iran. Moreover, we thank Clinical Research Development Unit of Poursina Hospital, Poursina Hospital, Guilan University of Medical Sciences, Rasht, Iran.
